# Ultrasonographic Validation of Anatomical Landmarks for Localization of the Tendon of the Long Head of Biceps Brachii

**DOI:** 10.1155/2017/1925104

**Published:** 2017-02-12

**Authors:** Saiyun Hou, John Harrell, Sheng Li

**Affiliations:** ^1^Department of Physical Medicine and Rehabilitation, Baylor College of Medicine, Houston, TX, USA; ^2^Department of Physical Medicine and Rehabilitation, University of Texas Health Science Center-Houston, Houston, TX, USA

## Abstract

*Objectives*. To establish anatomical landmarks for biceps tendon groove localization based on intrinsic anatomical relations and to validate the localization with ultrasonographic measurement.* Design*. Perspective, observational, single-blinded pilot study.* Participants*. 25 healthy male and female volunteers ages 24–50 years.* Methods*. We used two anatomical landmarks, the medial epicondyle vertical line related landmark and the coracoid process landmark. The distance from the groove skin mark to the medial epicondyle vertical line and the coracoid process was measured horizontally and was measured at 0° and 45° of shoulder external rotation, respectively.* Results*. Medial epicondyle vertical lines were 9.3 mm/21.5 mm medial to the groove at 0°/45° of shoulder external rotation, respectively. Correlation coefficients were 0.04/0.10, 0.32/0.42, and 0.26/0.37 for weight, height, and BMI in 0°/45° of shoulder external rotation, respectively. The distance between the coracoid process and the groove was 44.0 mm/62.2 mm in 0°/45° of shoulder external rotation, respectively. Correlation coefficients were 0.36/0.41, 0.36/0.54, and 0.18/0.12 for weight, height, and BMI in 0°/45° of shoulder external rotation, respectively.* Conclusions*. The medial epicondyle vertical line and the coracoid process landmark are both useful anatomical landmarks to localize the biceps groove. The anatomical landmark based localization is essentially not correlated with subject's weight, height, or BMI.

## 1. Introduction

The long biceps tendon arises mainly from the supraglenoid tubercle and partly from the superior glenoid labrum, passes through the glenohumeral joint, and enters the intertubercular groove. Its intra-articular and extra-articular portions are stabilized by a series of soft-tissue restraints arising from the coracohumeral ligament, superior glenohumeral ligament, and supraspinatus and subscapularis tendons [1]. Pathologies of the long head of the biceps tendon (LHBT) present an important source of shoulder pain, often causing significant losses in this joint flexion. Isolated disorders of LHBT include tendinopathy, dislocation, and partial or complete tears. LHBT tendinopathy is generally due to inflammatory, traumatic, and degenerative causes related to overuse, becoming chronic in most cases [2]. Also it is commonly accompanied by shoulder pathology such as rotator cuff dysfunction, scapular dyskinesis, adhesive capsulitis, glenohumeral joint arthritis, SLAP (superior labrum anterior to posterior) lesions, or supraspinatus tendinosis [3–5]. A few studies [6–9] have found a close relationship between rotator cuff tears and associated injuries produced in LHBT. Disorders of the LHBT are associated with rotator cuff tear in up to 90% of cases [6, 8]. Physical examination is unreliable in the diagnosis of LHBT pathology. Local anesthetic or steroid injection into the biceps long head tendon sheath is commonly used for diagnosis and treatment. Ultrasonography is preferred for visualizing the LHBT. However, it has a disadvantage of machine use and being time consuming. Long head of biceps palpation at the level of the intertubercular groove not only plays a central role in the physical examination of patients who present with anterior shoulder pain but is also the primary means of localization for biceps tendon sheath injections. Unfortunately, only approximately 5.3% overall accurate rate of long head of biceps groove palpation was reported in 25 examiners including attending physicians, fellows, and residents [10]. In this study we developed two anatomical landmarks, medial related vertical line, and coracoid related landmarks which are based on intrinsic anatomical relations. The relationship between those two landmarks and LHBT is consistent and reliable. The purpose of the present study was to introduce these two anatomical landmarks for localization of biceps tendon groove and to validate the localization with ultrasonographic measurement.

## 2. Methods

### 2.1. Design

The perspective, observational study was completed in a tertiary care center at an academic institution. All of participants were blinded to study. Two anatomical landmarks were developed to localize LHBT groove and localization was validated with ultrasonographic measurement.

### 2.2. Participants

Participants were recruited on a volunteer basis through word of mouth and posters. Participants were included if they are healthy adults between 18 and 64 years old. Exclusion criteria were those who (1) had a history of shoulder pain or injury and those of shoulder surgical intervention; of biceps tendinitis or injury; or of shoulder joint chronic pathology, including but not limited to such issues as osteoarthritis, Rheumatologic, or autoimmune disorders and (2) were pregnant or lactating. Each participant's height, weight, and calculated BMI were recorded.

### 2.3. Ethic Statement

The study protocol (H36580) was approved by the local Institutional Review Board and was conducted at a single-site rehabilitation center in an urban tertiary academic medical center. Written informed consent was obtained from all patients before enrollment into the study.

### 2.4. Equipment

All ultrasound scans were performed on a Philips iU22 ultrasound machine with a 12–5 MHz linear array transducer (Philips Ultrasound Systems, Bothell, WA). The measurement was performed with an electronic digital caliber (NEIKO TOOLS, Gardena, CA). A 90° angle rule was used to mark the site of medial epicondyle vertical line at intertubercular groove for each participant.

### 2.5. Procedures

Left shoulder was used to complete the study to standardize the procedure. After obtaining informed consent, age, weight, and height of the subject were recorded by a study coinvestigator. Two anatomical landmarks were used to localize the LHBT.

**Figure 1:**
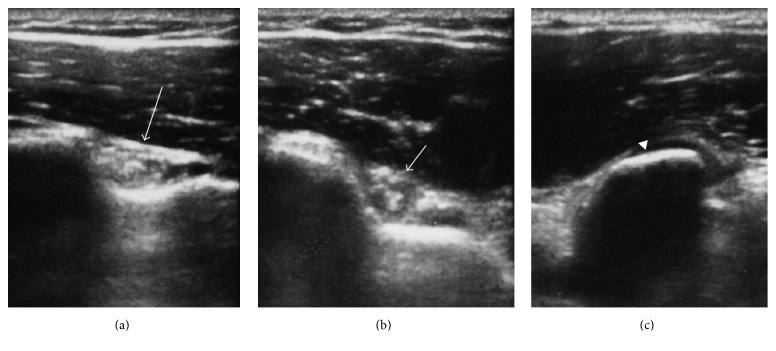
Biceps long head tendon groove (arrow) in the neutral position (a) and 45° of shoulder external rotation (b) was marked under ultrasound guidance. Coracoid process (arrowhead) was marked under ultrasound guidance (c).

**Figure 2:**
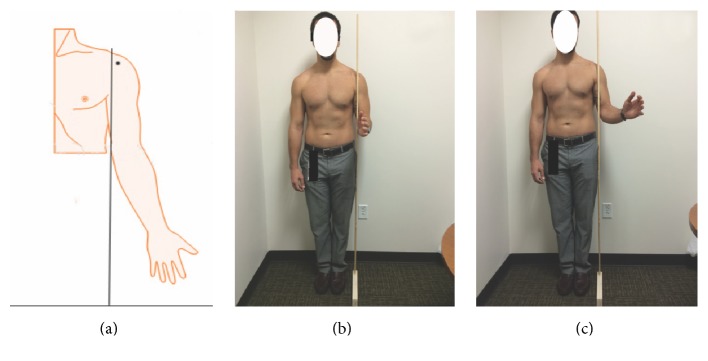
(a) Black line represents a vertical line passing through the medial epicondyle of humerus and perpendicular to ground. Black dot represents the intertubercular groove. Medial epicondyle related landmark was marked with a 90° angle ruler in the neutral position (b) and 45° of shoulder external rotation (c).

*(1) Medial Epicondyle Vertical Line Related Landmark*. Participants stood upright with the shoulder in the neutral position (0°), the elbow at 90° of flexion, and the forearm in the neutral position between pronation and supination. Firstly the biceps long head tendon groove was marked under ultrasound guidance (Figure 1(a)). Then a vertical line was drawn to pass through medial epicondyle of humerus and perpendicular to the ground with a 90° angle rule (Figure 2). The line to where the vertical line passed through anterior shoulder at the biceps groove level was marked. The distance from biceps long head tendon groove to the vertical line was measured horizontally with a digital caliper. Then participants changed their shoulder position to 45° of shoulder external rotation. The above procedure was repeated and the distance from biceps long head groove to the vertical line was measured again horizontally with a digital caliper (Figure 1(b)).

**Figure 3:**
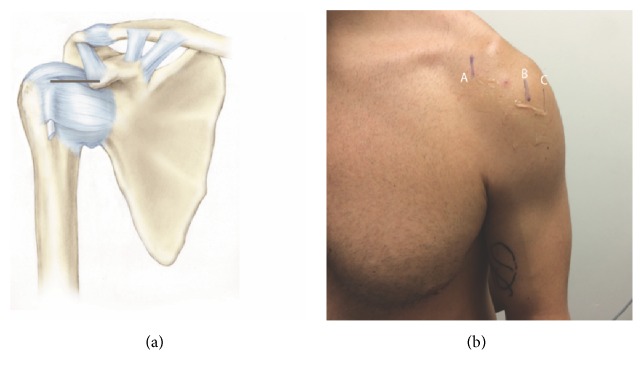
(a) Black line represents the distance between the coracoid process and the intertubercular groove. (b) Coracoid process (A), intertubercular groove in 0° (B), and 45° (C) of shoulder external rotation were marked with ultrasound. The distance of the coracoid process and the biceps long head tendon groove was measured horizontally between A and B or C at the coracoid level.

*(2) Coracoid Process Landmark*. Participants stood upright with the shoulder in the neutral position (0°), the elbow at 90° of flexion, and the forearm in the neutral position between pronation and supination. The coracoid process and biceps long head tendon groove were marked under ultrasound guidance (Figure 1(c)). The distance of the coracoid process and the biceps long head tendon groove was measured horizontally with a digital caliper at the coracoid level (Figure 3). The above procedure was repeated when participant's arm was kept at 45° of shoulder external rotation. The distance of the coracoid process and the biceps long head tendon groove was measured with a digital caliper at 45° of shoulder external rotation.

### 2.6. Data Analysis

Mean and 95% confidence interval (CI) of the distance measured at 0° and 45° of shoulder external rotation were calculated for those two anatomical landmarks, respectively. Also correlation coefficients between height, weight, and body mass index (BMI) and those two anatomical landmarks related measurement were calculated.

## 3. Results

25 healthy volunteers (16 female and 9 male; 24–50 years of age; mean, 29.5 ± 5.4 years) had body mass index (BMI) of 17.7–35.8 (mean, 23.2 ± 3.7). According to American CDC BMI classification criteria, this sample included 19 normal weight subjects (BMI 18.5–24.9), 1 underweight subjects (BMI < 18.5), and 5 overweight subjects (BMI 25–29.9).

All anatomic landmarks were identified under ultrasound guidance in all subjects. All medial epicondyle vertical lines were medial to the biceps groove at both 0° and 45° of shoulder external rotation. The horizontal distance between the biceps groove and medial epicondyle vertical line was found to be 9.3 mm (95% CI, 6.8 to 11.8) and 21.5 mm (95% CI, 18.9 to 24.1) in 0° and 45° of shoulder external rotation, respectively. Correlation coefficients were 0.04/0.10, 0.32/0.42, and 0.26/0.37 for weight, height, and BMI in 0°/45° of shoulder external rotation, respectively (Table 1).

The horizontal distance between the coracoid process and the biceps tendon groove was found to be 44.0 mm (95% CI, 41.5 to 46.5) and 62.2 mm (95% CI, 59.2 to 65.2) in 0° and 45° of shoulder external rotation, respectively. Correlation coefficients were 0.36, 0.36, and 0.18 for weight, height, and BMI in 0° neutral position, respectively. At 45° of shoulder external rotation, correlation coefficients were 0.41, 0.54, and 0.12 for weight, height, and BMI, respectively (Table 2). Collectively, the results support the intrinsic anatomical relations between these landmarks and the LHBT groove.

## 4. Discussion

The long head of the biceps tendon (LHBT) can be a source of pain or shoulder dysfunction [11]. Local anesthetic or steroid injection into the biceps long head tendon sheath is commonly used for diagnosis and treatment. Identifying the biceps tendon as a pain generator in patients presenting with shoulder pain syndromes has significant and diagnostic and therapeutic implications. An accurate and complete diagnosis facilitates optimal treatment [12].

In the study described herein, two anatomic landmarks for biceps tendon identification were used and their relationships to LHBT within the intertubercular groove were evaluated. The medial epicondyle vertical line lies approximately 1 centimeter medial to the biceps groove when the shoulder is in the neutral position and about 2 centimeters when shoulder is at 45° of external rotation. The coracoid process landmark lies approximately 4.4 centimeters when the shoulder is in the neutral position, about 6.2 centimeters when the shoulder is at 45° of external rotation. Therefore, these two anatomical landmarks are effective for LHBT identification and can be performed easily in an outpatient setting.

The relationship between the observed landmarks and the LHBT is rooted in intrinsic anatomical relations among them. The medial epicondyle of the humerus is a bony protrusion located on the medial side of distal humerus (Figure 2). The relationship among them is consistent and reliable. Given this intrinsic relation, this anatomical landmark based localization is not correlated with subject's weight, height, or BMI. The coracoid process is a small hook-like structure on the lateral edge of the superior anterior portion of the scapula. It is localized in the deltopectoral groove between the deltoid and pectoralis major muscles. There is certain intrinsic relation between coracoid process and intertubercular groove. Glenohumeral joint subluxation, dislocation, or effusion may change this relationship. Physician should be aware of it when patient has the comorbidity of GH joint pathology. Given this intrinsic relation, coracoid related landmark is not correlated with subject's weight or BMI. The height has the moderate correlation to the distance measured in 45° of shoulder external rotation, but no correlation in 0° of shoulder external rotation. Therefore we recommend that physicians localize the LHBT in the neutral position of shoulder when measuring with coracoid process related landmark.

The two anatomical landmarks introduced in this study were developed to identify the localization of LHBT within the intertubercular groove. The localization was validated with ultrasonographic measurement. Correlation between the distance and weight, height, and BMI increased when shoulder externally rotated to 45 degree compared with the neutral position for these two landmarks. We recommend that the neutral position of shoulder is a better way to localize the LHBT.

## 5. Limitations

Firstly, although 25 subjects were conducted for this pilot study, further study including increased sample size could help improve the accuracy of the measurements and increase its generalisability. Secondly, this measurement is reliable only if the coracoid process, LHBT, and medial epicondyle of humerus and LHBT are intact as was the case in our subjects. If the coracoid process and medial epicondyle of humerus are fractured or the LHBT is dislocated, these measurements cannot provide guidance about where the LHBT may be found. Thirdly, intraobserver and interobserver error were not included because all measurements were made by one author.

## 6. Conclusion

We used two new anatomical landmarks for biceps tendon groove localization. The medial epicondyle vertical line is a useful anatomical landmark to localize the biceps groove. It lies approximately 1 centimeter medial to the biceps groove when the shoulder is in the neutral position and about 2 centimeters when shoulder is at 45 degrees of external rotation. This anatomical landmark based localization is not correlated with subject's weight, height, or BMI. The coracoid process landmark is also a useful anatomical landmark to localize the biceps groove. It lies approximately 4.4 centimeters when the shoulder is in the neutral position, about 6.2 centimeters when the shoulder is at 45 degree of external rotation. No correlation exists between the distance to the biceps groove and weight, height, or BMI in 0° of shoulder external rotation, whereas a moderate correlation with height exists in 45° of shoulder external rotation. Those two anatomical landmarks allow for efficient and safe identification of the tendon of the long head of biceps brachii when examining or injection.

## Figures and Tables

**Table 1:** Medial epicondyle vertical line related landmark: average distance between the biceps groove and medial epicondyle vertical line were measured at 0° and 45° external rotation. Correlation coefficients between the distance and weight, height, and BMI were calculated at 0° and 45° of shoulder external rotation. CC: correlation coefficients. BMI: body mass index.

	Average distance (95% CI)	CC between the distance and weight	CC between the distance and height	CC between the distance and BMI
0° neural position	9.3 mm (6.8–11.8)	0.04	0.32	0.26
45° external rotation	21.5 mm (18.9–24.1)	0.10	0.42	0.37

**Table 2:** Coracoid process landmark: average distance between biceps groove and coracoid process were measured at 0° and 45° external rotation. Correlation coefficients between the distance and weight, height, and BMI were calculated at 0° and 45° external rotation. CC: correlation coefficients. BMI: body mass index.

	Average distance (95% CI)	CC between the distance and weight	CC between the distance and height	CC between the distance and BMI
0° neural position	44.0 mm (41.5–46.5)	0.36	0.36	0.18
45° external rotation	62.2 mm (59.2–65.2)	0.41	0.54	0.13

## Abbreviations

BMI: Bone mass index
CI: Confidence interval
LHBT: Long head of the biceps tendon
GH: Glenohumeral joint
SLAP: Superior labrum anterior to posterior.

## References

[1] Habermeyer P., Magosch P., Pritsch M., Scheibel M. T., Lichtenberg S. (2004). Anterosuperior impingement of the shoulder as a result of pulley lesions: A Prospective Arthroscopic Study. *Journal of Shoulder and Elbow Surgery*.

[2] Longo U. G., Loppini M., Marineo G., Khan W. S., Maffulli N., Denaro V. (2011). Tendinopathy of the tendon of the long head of the biceps. *Sports Medicine and Arthroscopy Review*.

[3] Khazzam M., George M. S., Churchill R. S., Kuhn J. E. (2012). Disorders of the long head of biceps tendon. *Journal of Shoulder and Elbow Surgery*.

[4] Harwood M. I., Smith C. T. (2004). Superior labrum, anterior-posterior lesions and biceps injuries: diagnostic and treatment considerations. *Primary Care: Clinics in Office Practice*.

[5] Peltz C. D., Perry S. M., Getz C. L., Soslowsky L. J. (2009). Mechanical properties of the long-head of the biceps tendon are altered in the presence of rotator cuff tears in a rat model. *Journal of Orthopaedic Research*.

[6] Beall D. P., Williamson E. E., Ly J. Q. (2003). Association of biceps tendon tears with rotator cuff abnormalities: degree of correlation with tears of the anterior and superior portions of the rotator cuff. *American Journal of Roentgenology*.

[7] Chen C.-H., Hsu K.-Y., Chen W.-J., Shih C.-H. (2005). Incidence and severity of biceps long head tendon lesion in patients with complete rotator cuff tears. *Journal of Trauma—Injury, Infection and Critical Care*.

[8] Murthi A. M., Vosburgh C. L., Neviaser T. J. (2000). The incidence of pathologic changes of the long head of the biceps tendon. *Journal of Shoulder and Elbow Surgery*.

[9] Peltz C. D., Hsu J. E., Zgonis M. H., Trasolini N. A., Glaser D. L., Soslowsky L. J. (2010). The effect of altered loading following rotator cuff tears in a rat model on the regional mechanical properties of the long head of the biceps tendon. *Journal of Biomechanics*.

[10] Gazzillo G. P., Finnoff J. T., Hall M. M., Sayeed Y. A., Smith J. (2011). Accuracy of palpating the long head of the biceps tendon: an ultrasonographic study. *PM&R*.

[11] Elser F., Braun S., Dewing C. B., Giphart J. E., Millett P. J. (2011). Anatomy, function, injuries, and treatment of the long head of the biceps brachii tendon. *Arthroscopy—Journal of Arthroscopic and Related Surgery*.

[12] Zanetti M., Weishaupt D., Gerber C., Hodler J. (1998). Tendinopathy and rupture of the tendon of the long head of the biceps brachii muscle: evaluation with MR arthrography. *American Journal of Roentgenology*.

